# Field Evaluation of a Prototype Paper-Based Point-of-Care Fingerstick Transaminase Test

**DOI:** 10.1371/journal.pone.0075616

**Published:** 2013-09-30

**Authors:** Nira R. Pollock, Sarah McGray, Donn J. Colby, Farzad Noubary, Huyen Nguyen, The Anh Nguyen, Sariah Khormaee, Sidhartha Jain, Kenneth Hawkins, Shailendra Kumar, Jason P. Rolland, Patrick D. Beattie, Nguyen V. Chau, Vo M. Quang, Cori Barfield, Kathy Tietje, Matt Steele, Bernhard H. Weigl

**Affiliations:** 1 Division of Infectious Diseases, Beth Israel Deaconess Medical Center, Boston, Massachusetts, United States of America; 2 Department of Laboratory Medicine, Boston Children’s Hospital, Boston, Massachusetts, United States of America; 3 PATH, Seattle, Washington, United States of America; 4 Department of Medicine, Division of General Medicine & Primary Care, Beth Israel Deaconess Medical Center, Boston, Massachusetts, United States of America; 5 Harvard Medical School AIDS Initiative Vietnam, Ho Chi Minh City, Vietnam; 6 Institute for Clinical Research and Health Policy Studies, Tufts Medical Center, and Tufts Clinical and Translational Science Institute, Tufts University, Boston, Massachusetts, United States of America; 7 Center for Applied Research on Men and Health, Ho Chi Minh City, Vietnam; 8 Harvard-MIT Health Sciences and Technology Program, Harvard Medical School, Boston, Massachusetts, United States of America; 9 Diagnostics For All, Cambridge, Massachusetts, United States of America; 10 Hospital for Tropical Diseases, Ho Chi Minh City, Vietnam; Gentofte University Hospital, Denmark

## Abstract

Monitoring for drug-induced liver injury (DILI) via serial transaminase measurements in patients on potentially hepatotoxic medications (e.g., for HIV and tuberculosis) is routine in resource-rich nations, but often unavailable in resource-limited settings. Towards enabling universal access to affordable point-of-care (POC) screening for DILI, we have performed the first field evaluation of a paper-based, microfluidic fingerstick test for rapid, semi-quantitative, visual measurement of blood alanine aminotransferase (ALT). Our objectives were to assess operational feasibility, inter-operator variability, lot variability, device failure rate, and accuracy, to inform device modification for further field testing. The paper-based ALT test was performed at POC on fingerstick samples from 600 outpatients receiving HIV treatment in Vietnam. Results, read independently by two clinic nurses, were compared with gold-standard automated (Roche Cobas) results from venipuncture samples obtained in parallel. Two device lots were used sequentially. We demonstrated high inter-operator agreement, with 96.3% (95% C.I., 94.3–97.7%) agreement in placing visual results into clinically-defined “bins” (<3x, 3–5x, and >5x upper limit of normal), >90% agreement in validity determination, and intraclass correlation coefficient of 0.89 (95% C.I., 0.87–0.91). Lot variability was observed in % invalids due to hemolysis (21.1% for Lot 1, 1.6% for Lot 2) and correlated with lots of incorporated plasma separation membranes. Invalid rates <1% were observed for all other device controls. Overall bin placement accuracy for the two readers was 84% (84.3%/83.6%). Our findings of extremely high inter-operator agreement for visual reading–obtained in a target clinical environment, as performed by local practitioners–indicate that the device operation and reading process is feasible and reproducible. Bin placement accuracy and lot-to-lot variability data identified specific targets for device optimization and material quality control. This is the first field study performed with a patterned paper-based microfluidic device and opens the door to development of similar assays for other important analytes.

## Introduction

There is great need for high-quality, low-cost, point-of-care (POC) diagnostics that can increase access to testing and improve patient care in resource-limited settings. An important example of inadequate access to testing in resource-limited settings is monitoring for drug-induced liver injury (DILI) in patients on potentially hepatotoxic medications. In resource-rich settings, serial monitoring for DILI via measurements of serum transaminases (aspartate aminotransferase [AST] and alanine aminotransferase [ALT]) in at-risk patients (particularly those with underlying liver disease) is a standard part of medical care. However, this monitoring is often limited or unavailable in resource-limited settings. Monitoring for DILI is particularly relevant for patients on tuberculosis (TB) and/or HIV therapy [1], [2], and thus lack of access to this testing is particularly problematic in the resource-limited areas most affected by these diseases. Transaminase monitoring typically involves collecting whole blood by venipuncture, centrifuging to separate serum or plasma, and testing that serum/plasma on a large automated platform. Such systems require highly trained technicians for maintenance and are quite expensive, impacting test availability in resource-limited settings. If performed, testing is often done in centralized or regional laboratories, lengthening result turn-around times. Because of these obstacles, in many resource-limited settings patients on potentially hepatotoxic medications receive minimal or no monitoring during treatment.

To advance towards the ultimate goal of providing universal access to affordable POC DILI screening, Pollock and Rolland et al. have recently described development and early clinical testing of a paper-based, multiplexed, microfluidic assay designed for rapid, semi-quantitative, and visual measurement of AST and ALT in a fingerstick specimen [3]. This device is a representative of an emerging class of paper-based microfluidic platforms [4]–[20]. There is currently great interest in the potential diagnostic utility of microfluidic platforms based on paper, given the benefits of portability, disposability, lack of power and instrumentation requirements, and extremely low cost. Paper-based microfluidic devices consist of hydrophilic paper channels defined by patterning of hydrophobic barriers (e.g., [3], [4], [6], [7], [18], [20]) or by cutting (e.g., [10]–[14], [17]). Using these defined channels, fluid flow (drawn by wicking) can be directed towards specific detection zones and operations such as filtration, mixing, and splitting can be performed autonomously. Proof-of-principle studies [5]–[18], [20] have demonstrated the ability to conduct clinical chemistry, enzymatic, and immunoassay tests on patterned paper, visually and quantitatively (the latter through the use of cell phone cameras), and thus have demonstrated the potential for clinical application of paper-based microfluidic technology. The work by Pollock and Rolland et al. [3] advanced this field by presenting the first validation of a paper-based microfluidic device using real clinical specimens and the first demonstration of a field-ready prototype clinical test for transaminase monitoring. That study showed that the paper-based assay could, in 15 minutes, provide visual measurements of AST and ALT in whole blood or serum which allowed the user to place those values into one of three readout “bins” (<3x upper limit of normal [ULN], 3–5x ULN, and >5x ULN, corresponding to familiar clinical thresholds for TB and HIV treatment monitoring, e.g. [1], [2]) with >90% accuracy as compared to automated methods [3].

Despite this large body of proof-of-principle work in the field of paper-based microfluidics, there has yet to be an actual field study showing that a clinical diagnostic assay utilizing this platform technology actually works for real-time clinical testing in a target clinical population and setting. Note that we distinguish paper-based microfluidic tests from lateral flow tests, which are widely used in current clinical practice (including at POC). Lateral flow tests are also considered “paper-based devices” in that they utilize absorptive material strips. However, they typically are limited to immunoassays and run small numbers of tests (1 to 2) in series (requiring that reagents and buffers for each test be compatible and that assays not cross-react). In contrast, paper-based microfluidic devices, similarly to their conventional plastic-based microfluidic counterparts, can be designed to integrate many analytical functions and perform assays based on a wide range of assay principles [19], [21]. As discussed in this paper, they can also split a single, low-volume (<40 µl) sample into multiple separate streams, each of which can be assayed in parallel. This avoids cross-reactivity between assays and allows for high-level multiplexing of independently optimized assays.

We here present the results of the first fingerstick evaluation of an ALT-only version of the paper-based transaminase test in 600 patients undergoing HIV treatment in a single clinic in Vietnam. The goals of this study were to assess operational feasibility, inter-operator variability, lot-to-lot variability, device failure rate, and device accuracy, with the intention to utilize results to modify the device for further field testing as needed. Our results, obtained in a target clinical population and environment, as performed by local health care workers, indicate that the device operation and reading process is both feasible and reproducible, thus answering a major question about the potential usability of this type of device. Bin placement accuracy data and lot-to-lot variability analysis identified specific targets for device optimization and material quality control.

## Materials and Methods

### Device Production

Devices were fabricated as previously reported [3] and pouched individually in foil-lined bags with one (1 gm) packet of silica (Electron Microscopy Sciences, Hatfield, PA) per bag. Two device lots (LFT042412 and LFT061312) were produced and used for this study. These devices had a shelf-life of five months if stored at 35°C, as estimated from accelerated stability data.

### Device Shipment and Storage

Each of the two device lots was shipped (via FedEx) from Boston to Ho Chi Minh City and stored at ambient temperatures, to approximate the probable conditions of distribution of a commercially available device. Temperatures during shipment were recorded for Lot 2 using a temperature monitor (TinyTag Talk 2 Temp Logger TK-4014), and the resulting profile used as a proxy for the Lot 1 shipment (which did not include a data logger). Ambient temperatures during study enrollment were monitored using a temperature and humidity data logger (Extech®) set to record at 15-minute intervals. Historical temperature data (daily minimum, maximum, and mean) from Weather Underground (wunderground.com) was used as a proxy for ambient temperatures in the clinic during periods when no data logger data was available (between pilot and study start, and over two weekends at the start of enrollment). A comparison of five daily measurements (minimum, maximum, mean) from the clinic data logger and Weather Underground confirmed agreement of the two sources of temperature data.

### Study Setting and Population

Subjects were recruited from the outpatient HIV clinic of the Hospital for Tropical Diseases (HTD), Ho Chi Minh City, Vietnam. Eligible patients were adults (≥18 years old) receiving HIV treatment through the HTD clinic who were scheduled to receive routine ALT monitoring on the day of enrollment, willing to undergo a fingerstick in addition to routine care, and able to provide informed consent.

### Study Training Procedures

Training was conducted in conjunction with preparation for the pilot study, approximately one month prior to study start, with proficiency tests immediately before the start of both the pilot and evaluation studies. Training was tailored for the purposes of this study and was provided by representatives of the sponsor (PATH) and the device manufacturer (Diagnostics For All [DFA]), who offered intensive, individualized instruction to each nurse. The training curriculum included review of the study objectives and recruitment procedures, overview of the device structure and function, steps for completing the fingerstick and transferring sample to the device per the manufacturer’s product insert, and practice reading with mock devices (with various specific ALT values and control results represented in the detection zones, generated using scanned images of completed devices). In addition, nurses were specifically instructed about the study requirement to read and record device results privately, without interaction with any other individual. The study nurses were required to pass a proficiency test using the mock devices (pass criteria: ≥80% bin placement accuracy and 100% determination of invalids) before patient enrollment could start. If they failed this test, they were retrained with the mock devices and given another test. Each nurse was allowed a maximum of two trials to pass the test. During the pilot phase (see below), the study nurses received immediate feedback on correct and incorrect use (including fingerstick, sample transfer procedure, and device reading) from an expert user (DFA representative). No additional training or feedback was given once evaluation study enrollment began.

### Study Protocol

All tests were performed as per a product insert provided by the manufacturer (DFA, Cambridge, MA, USA). After wiping fingers with alcohol swabs, fingersticks were performed with safety lancets (SurgiLance® SLN 300, MediPurpose, Duluth, GA, USA) and blood collected with commercially available 35-µL capillary tubes (Microsafe, SafeTec LLC, Ivyland, PA, USA). Devices were incubated for 12 to 14 minutes as per the product insert (time dependent on ambient temperature) in open petri dishes in a separate incubation area for safety. These petri dishes were cleaned daily with a bleach solution and replaced weekly. Following the allocated incubation period, each device was read in quick succession by two nurses, defined by role as N1 (who conducted the fingerstick and sample transfer to the device) and N2 (who set the timer for the incubation period and moved the petri dish to the device incubation area.) (Note: the N1 role was filled by one of the three trained study nurses for the entire study; the other two study nurses took turns filling the N2 role). N2 typically (but not always) read the device first, followed by N1, who had the more time-consuming task of performing the fingersticks and managing patient flow. The nurses were specifically instructed not to communicate during the reading procedure and adhered to this procedure, preserving the independence of the two readings. Each device was scanned immediately after visual reading (Canon Inkjet Photo All-in-One PIXMA MP287, Canon Inc., Tokyo, Japan). Neither the patients nor their doctors were informed of the results of their fingerstick testing.

Automated ALT testing was performed in parallel (Roche Cobas® ALT assay without pyridoxal phosphate activation, run on a Roche Cobas® 6000 platform with C311 and C501 analyzer modules) using blood obtained by venipuncture (venipuncture was performed prior to fingerstick). Per clinic and laboratory routine, blood was sent to the HTD clinical laboratory within one to two hours after draw and separated by centrifugation to generate plasma for AST/ALT testing on the Roche Cobas® platform per the manufacturer’s protocol (Roche Diagnostics, Indianapolis, IN, USA). Specimens went on the machine for testing within 30 minutes of receipt in the laboratory. Results obtained using this method are in U/L, with normal reference ranges of 0 to 40 U/L (male) and 0 to 33 U/L (female).

Additional clinical and laboratory data was collected as available for each subject, including hepatitis B virus (HBV) status, hepatitis C virus (HCV) status, current HIV medications, current TB medications (if any), and most recent CD4 count/date. Results of any laboratory tests ordered concurrently with ALT on the day of enrollment were also captured as available (specifically including AST, hemoglobin, hematocrit, platelet count, creatinine, and CD4).

A pilot study (50 subjects) was performed first to assess operational feasibility of the device and to confirm that the study procedures were working as expected in the clinic environment. Following the pilot, enrollment for the evaluation study (600 subjects) commenced. Data from the pilot study was not combined with data from the evaluation study, and data from the pilot study is not reported in this manuscript. The sample size for the evaluation study was driven by numbers of subjects needed to enroll in order to ultimately obtain at least 20 fingerstick tests from subjects with gold-standard ALT values falling in the upper bin (>5x ULN).

### Human Subjects Protections/Ethics Statement

This study was approved by the PATH Research Ethics Committee and by the respective Institutional Review Boards of the Hospital for Tropical Diseases and Beth Israel Deaconess Medical Center. All subjects provided written informed consent.

### Statistical Analysis

All data analysis was conducted using R (http://www.R-project.org). Binary results were compared using proportions with exact confidence intervals, and inter-reader agreement was assessed using the joint probability of agreement, diagonal plots, and the intraclass correlation coefficient (ICC).

## Results

### 3-zone Paper-based Transaminase Test: Design, Production, and Storage

**Figure 1:**
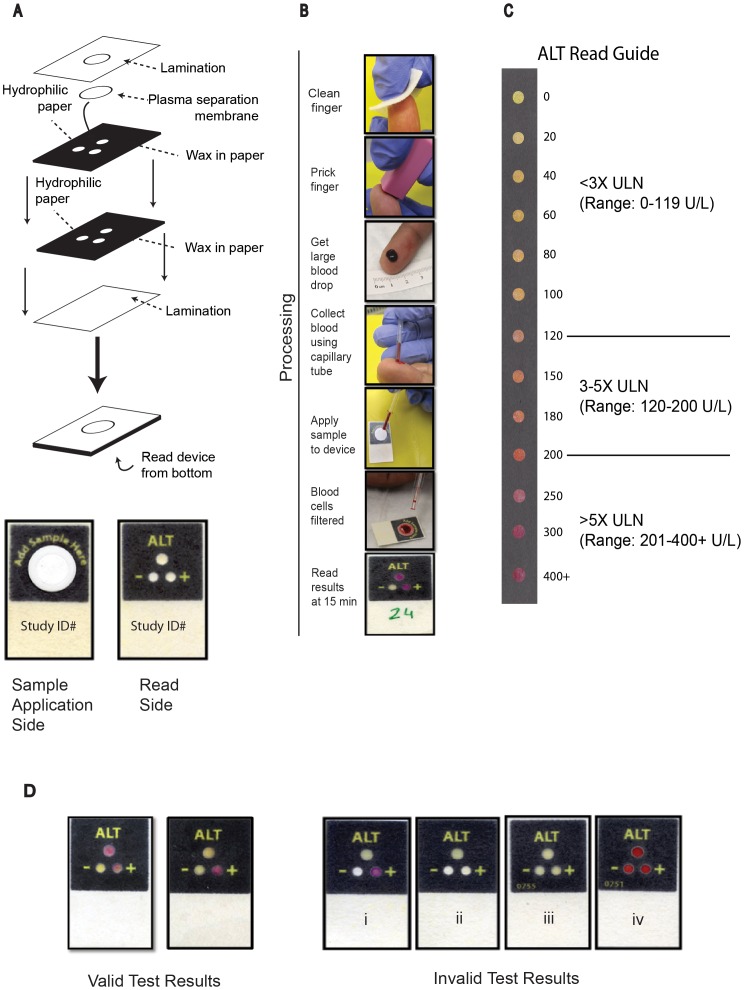
Schematic of 3-zone paper-based transaminase test design and performance. (A) The paper-based transaminase test is made from two layers of similarly patterned-paper, a plasma separation membrane (PSM), and lamination to protect the device from the environment. (A and B) A hole in the lamination allows for a fingerstick blood sample (collected using a capillary tube) to be applied to the PSM; blood cells are captured and retained by the PSM while plasma wicks into the individual “zones” in the first layer of paper below. In those zones, the plasma fluid reconstitutes dried reagents (as required for the zone-specific chemistry) and continues to wick to the second layer of paper, where analytes in the plasma react with additional dried reagents in each detection zone and generate visual colorimetric signals. (B and C) After a total of approximately 12 to 14 minutes incubation at ambient temperature, the color in the ALT test zone can be interpreted and semi-quantified using a visual “read guide” (C). The results of the test are interpreted as being within one of the following three “bins”: <3x the upper limit of normal (ULN) (0–119 U/L), 3–5x ULN (120–200 U/L), or >5x ULN (>200 U/L). (D) 2 additional control zones notify the user of invalid results. The negative control zone (marked “−”) allows assessment of insufficient sample volume (i, ii, zone white rather than yellow) or hemolysis (iv, zone red rather than yellow), while the positive control zone (marked “+”) allows assessment of damaged reagents (iii, zone not red); each zone is interpreted as “valid” or “invalid”. A result of “invalid” in either of the two control zones invalidates the entire device. (ALT = alanine aminotransferase. ULN = upper limit of normal).

DFA’s postage-stamp-sized (33×20×0.5 mm), 3D device is made by layering patterned paper (Figure 1A). To create each layer of patterned paper, a wax-based printer and a heat source are used to print hydrophobic barriers into a sheet of paper in order to create microfluidic, hydrophilic paths within the paper, through which flow (drawn by wicking) can be directed to specific “detection zones”. Layers of patterned paper can be stacked to generate 3D devices by depositing patterned layers of hydrophobic adhesive via screen printing and adhering multiple sheets together. This three-zone device enables performance of three measurements on a single fingerstick sample: one zone measures ALT and two control zones ensure proper device performance (Figure 1A). Each of the three zones has a unique environment (reagents, buffers, pH) that ensures specificity. Fingerstick blood is collected using a lancet and capillary tube (Methods) and applied directly to the device (Figure 1B). The ALT assay, based on peroxidase chemistry ([3]; of note, the assay does not include pyridoxal phosphate activation), generates a red dye in the presence of elevated ALT levels. The test was carefully engineered for visual readout, such that the ALT test zones provide a strong color change across the target clinical range (Fig. 1C). The color change was optimized to correspond to the cutoffs currently used for clinical management decisions per TB treatment guidelines in the United States [1]. Thus the results of the test are visually interpreted as being within one of the following three “bins”: <3x the ULN (0–119 U/L), 3–5x ULN (120–200 U/L), or >5x ULN (>200 U/L). Using the additional color gradation within each bin on the read guide, the reader can approximate where within the bin the result falls, allowing semi-quantitative readout (Figure 1C). The control zones notify the user of insufficient sample volume or hemolysis (viewed in the negative control zone), or damaged reagents (viewed in the positive control zone); each zone is interpreted as “valid” or “invalid” and an “invalid” result in any control zone invalidates the entire device (Figure 1D).

Devices were produced at DFA (Cambridge, MA, USA) and shipped to Ho Chi Minh City, Vietnam, where they were stored at ambient temperature (Methods), primarily in the study clinic. Two lots of devices were manufactured and were used sequentially for the study (Lot 1 for the first 218 subjects; Lot 2 for the next 382 subjects). Each lot of devices spent approximately eight weeks in storage in ambient conditions and all devices were used at least eight weeks before expiration dates (based on storage at 35°C) provided by the manufacturer. Daily temperatures in Ho Chi Minh City (°C, Methods) during storage of each lot prior to use in the study ranged from a minimum of 22–27 (average: 24.3) to a maximum of 30–36 (average: 33.5) for Lot 1 devices and from a minimum of 23–26 (average: 23.7) to a maximum of 31–34 (average: 33.1) for Lot 2 devices. Temperature ranges (°C) measured in the clinic during study testing (via a temperature logger) were 27.8–32.5 (average: 30.3) for Lot 1 and 28.3–32.7 (average: 29.9) for Lot 2. Comparison of maximum ambient city temperatures to maximum temperatures measured in the clinic on study testing days indicated that maximum city temperatures did not underestimate the temperature maximums the devices experienced in the clinic. Humidity ranges during study testing (%) were 55.9–85.2 (average: 70.9) for Lot 1 and 54.4–83.3 (average: 72.7) for Lot 2.

### Setting and Study Participants

The study was performed in the outpatient HIV clinic of the Hospital for Tropical Diseases (HTD) in Ho Chi Minh City, Vietnam. This clinic has approximately 3,000 HIV-positive patients on treatment provided free through the Vietnam Ministry of Health. A significant proportion are on Nevirapine-containing regimens (known to confer risk of DILI) and co-infection with HBV (15% prevalence) and/or HCV (25% prevalence) is common (unpublished data, HTD). The clinic also has an existing practice of routine transaminase monitoring (once every six months) for patients receiving HIV treatment, following Vietnamese national guidelines [22]. Eligible subjects (Methods) were recruited by their physicians during routine clinic visits. To ensure enrollment of sufficient subjects with ALT values ultimately falling into the highest two bins (3–5x ULN and >5x ULN), patients with known HBV, known HCV, or history of past elevated transaminases (≥90 U/L) were prioritized for enrollment. Only patients scheduled for routine clinical ALT testing (specimen collected by venipuncture) by their physicians were recruited to the study; after venipuncture, subjects proceeded to fingerstick collection.

All participants were ambulatory, enrolled in the free public anti-retroviral therapy (ART) program at the hospital, and on HIV treatment at the time of enrollment. The median age was 32 years (range 20 to 62) and 83% were male. Median CD4 count (most recent measurement) was 277.5 cells/mm^3^ (inter-quartile range 199–397). Almost all subjects were on first-line ART; the most common regimens taken were zidovudine/lamivudine/nevirapine (32%), zidovudine/lamivudine/efavirenz (27%), and tenofovir/lamivudine/efavirenz (18%). Only 3% of subjects were on second-line ART containing lopinavir/ritonavir.

### Pre-study Training

Training was intensive and individually tailored, as detailed above (Methods); complete operational data collected during this training will be reported separately. Training was conducted prior to the 50-subject pilot phase, and nurses were required to pass a proficiency test prior to beginning that pilot phase and a second test prior to start of evaluation phase enrollment. Despite passing the proficiency test prior to the pilot, expert observers determined that the nurses required (and thus were given) additional guidance during the pilot to correctly execute the sample transfer procedure and later interpret the device readout, indicating that mock device use during training was potentially insufficient without real-time feedback during actual patient testing. By the completion of the pilot, expert monitors observed that all three study nurses were comfortable and proficient with both sample transfer and device interpretation. The pre-evaluation phase proficiency test and a study monitoring visit during the evaluation phase confirmed skills retention.

### Testing Protocol and Adherence

After completion of the 50-subject pilot study (Methods), the paper-based ALT test was performed at POC on fingerstick blood samples from 600 patients using a product insert provided by the manufacturer, a safety lancet, and a 35-µL capillary tube (Methods, Figure 1). A timer was set for an allotted device incubation time as determined by the ambient temperature (20–24°C, incubate 18 minutes; 25–29°C, 14 minutes; 30–33°C, 12 minutes; 34–37°C, 10 minutes; per the product insert, reading the device within a 10 minute window after this incubation time was acceptable.). When the timer went off (“set time”), the device was read independently by each of two clinic nurses in turn, using the read guide provided (Figure 1C); nurses were asked to independently record both a result “bin” (<3x, 3–5x, or >5x ULN) and an absolute value (U/L, rounded to the nearest 10 U/L) for ALT results. Nurses also evaluated the two control spots to determine whether the test was valid or invalid, following the product insert (Methods, Figure 1D). The two nurses did not communicate with each other during their reading. Temperature and humidity at the time of testing were recorded as noted above. After completion of visual reading, each device was digitally scanned (Methods) to preserve data for subsequent expert interpretation (described below).

All 600 testing events generated visually interpretable data. There were no mismatches between measured ambient temperature (via the clinic logger) and the setting of the timer as per the guidelines in the product insert. The majority of devices were read within 2 minutes of the set time by both nurses, and all devices were read well within the 10-minute window allowed per the product insert. For Nurse 1, only 45/600 devices were read more than two minutes after the set time (32/45 were read three minutes after, 9/45 were read four minutes after, 1/45 was read five minutes after, and 3/45 were read six minutes after the set time), and for Nurse 2, 13/600 devices were read greater than two minutes after the set time (9/13 were read three minutes after, 3/13 were read four minutes after, and 1/13 was read six minutes after the set time).

### Device Performance

#### Validity analysis

Two “expert readers” (one from PATH, and one from DFA, both blinded to results of the nurses’ visual reads) independently reviewed the scanned images to determine whether each device was valid or invalid and, if invalid, why (negative control failure, positive control failure, or failure of both controls [Figure 1D]). All 600 scanned images were interpretable. However, two devices and all associated data had to be excluded from analysis due to enrollment error (one subject was not currently taking HIV medications and thus did not meet inclusion criteria, and the other was accidentally enrolled twice), leaving 598 device results available for analysis (218 from Lot 1, and 380 from Lot 2.) 13/598 device results were read discordantly by the two expert readers (11/13 were called invalid by only Expert Reader 1, and 2/13 were called invalid by only Expert Reader 2). Notably, the majority of this discordance was due to disagreement about the presence or absence of visible hemolysis in the negative control zone (Figure 1D, iv). To resolve this discordance, a third expert reader (from DFA; also blinded to all other study results) was asked to read a set of images including the discordant ones. The result (valid or invalid) agreed upon by two of the three expert readers was used as the final classification for each device.

After resolution of these discordant results, there were a total of 57 devices classified as invalid and 541 devices classified as valid (overall invalid rate: 57/598 [9.5%]); these validity classifications were subsequently used as the gold standard against which to compare validity results obtained by the nurses reading the devices in real time. Of these 57 invalid results, 52/57 were due to negative control “failure” (specifically, hemolysis visible in the negative control well; Figure 1D, iv), leading to an overall rate of invalids due to hemolysis of 8.7% (52/598). Of these 52 devices that were invalid due to hemolysis, 46 were devices from Lot 1 (46/218 = 21.1% hemolysis rate) and 6 were devices from Lot 2 (6/380 = 1.6% hemolysis rate). Review of materials used in the two device lots revealed that different lots of plasma separation membranes had been used to make the two device lots, suggesting that this material was the basis of the substantial lot-to-lot variability in hemolysis rates. 4/57 invalids were due to positive control failure (see Figure 1D, iii; overall rate 0.7% [4/598]) and 1/57 invalids were due to negative control failure (i.e., failure to “activate” due to insufficient sample volume, Figure 1D, i and ii; overall rate 0.2% [1/598]). There were no devices for which more than one control zone “failed”.

Gold-standard validity classifications were compared to the classifications made by Nurse 1 and Nurse 2 (n = 598 for Nurse 1, and n = 597 for Nurse 2 (Nurse 2 did not score validity for one device)). Nurse 1 validity classifications showed 93.3% (91.0–95.2) agreement with gold standard classifications, and Nurse 2 results showed 90.6% (88.0–92.8) agreement with gold-standard classifications. The majority of the disagreement with the gold standard for both nurses was due to a gold-standard “invalid” classification versus a “valid” classification made by the nurse, and specifically due to disagreement about the presence or absence of visible hemolysis, suggesting that the expert readers (in review of the scanned images) were seeing hemolysis that the nurses did not see in real time. Nonetheless Nurse 1 and Nurse 2, both reading in real time, agreed with each other on 571/597 classifications (95.6% [93.7–97.1]).

#### Device accuracy

**Figure 2:**
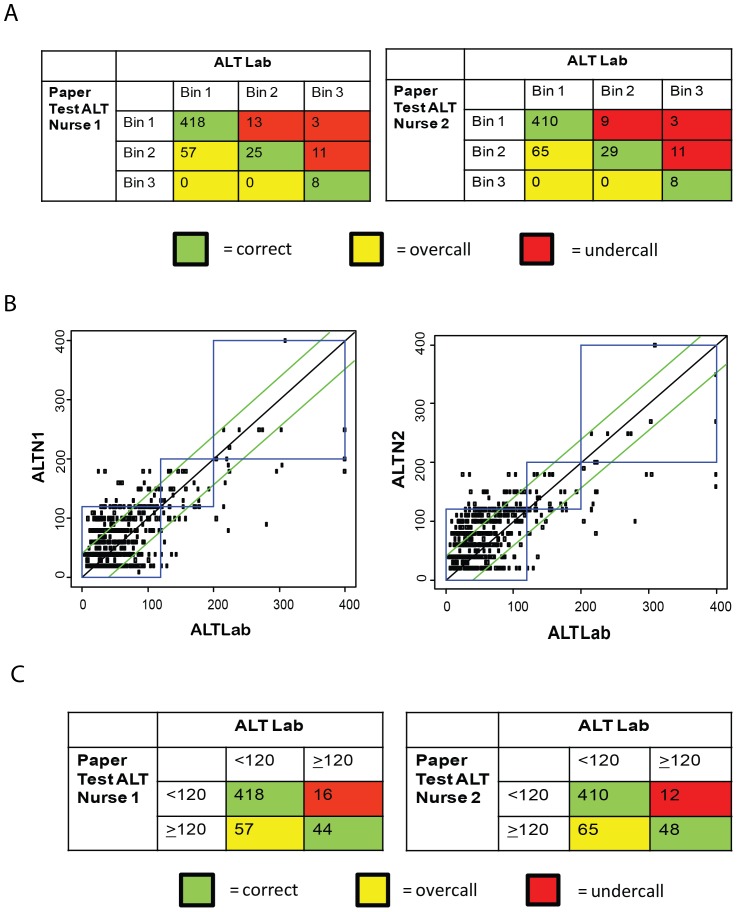
Comparison of paper-based transaminase test results to automated test results. (A) Bin placement accuracy. Nurse 1 (left table) and Nurse 2 (right table) visual device results (classified by “bin,” i.e. Bin 1 = <3x ULN [0–119 U/L], Bin 2 = 3–5x ULN [120–200 U/L], and Bin 3 = >5x ULN [>200 U/L]) were compared with automated device results (ALT Lab, also classified by bin) as the gold standard. Colored boxes indicate result pairs in which the visual result was in the same bin as the automated result (green, “correct”), a higher bin than the automated result (yellow, “overcall”), or a lower bin than the automated result (red, “undercall”). Per-bin accuracy for visual reading (Results) was calculated by dividing the number of correctly binned samples in each bin by the total number of samples in that bin. Overall bin placement accuracy (Results) was defined as the overall percentage of visual device results which fell in the same bin as the paired automated result. (B) Direct comparison of Nurse 1 (left plot, ALTN1) and Nurse 2 (right plot, ALTN2) visual device results to automated test results (ALTLab) for each subject. In each plot, the black diagonal line corresponds to the line of equality; the green diagonal lines correspond to +/−40 U/L from the line of equality; and the blue boxes represent ALT bins within which values for both the paper-based device and the automated method are within the same range: <3x ULN (0–119 U/L), 3 to 5x ULN (120–200 U/L), or >5x ULN (>200 U/L). (C) Reanalysis of the bin placement results (Nurse 1, left table; Nurse 2, right table) using a binary cutoff creating only two bins (<120 U/L = low, ≥120 U/L = high), as might be used in a “triage” or “screening” use scenario. (ALT = alanine aminotransferase. ULN = upper limit of normal).

All 57 devices with gold-standard invalid classification were excluded from further analysis, leaving 541 devices and associated data for analysis of device accuracy. Additionally, 6/598 devices were excluded from analysis because automated ALT testing of venipuncture blood had not been performed (due to clerical error), leaving no automated result to compare to the paper-based test. After these exclusions, there were 535 paper device and automated ALT result pairs available for analysis. Our primary measure was bin placement accuracy, which was defined as a visual device result which fell in the same “bin” (<3x ULN [<120 U/L], 3–5x ULN [120–200 U/L], or >5x ULN [>200 U/L]) as the automated result. In a small number of cases, a nurse assigned the wrong bin to a visual result (e.g., a result of 200 U/L had been assigned to Bin 3 rather than Bin 2); these mismatches were corrected prior to data analysis. The bin distribution of true ALT values was as follows: 475 results fell in Bin 1, 38 results in Bin 2, and 22 results in Bin 3 (total 535). Results of bin placement analysis are shown in Figure 2A. Per-bin accuracy was highest in Bin 1 (88.0/86.3% for Nurse 1 and Nurse 2, respectively) and lower in Bin 2 (65.8/76.3%) and Bin 3 (36.4/36.4%). Overall bin placement accuracy for Nurse 1 was 84.3% (80.9–87.3) and for Nurse 2 was 83.6% (80.1–86.6). A direct comparison of Nurse visual-read ALT results (reported during reading to the nearest 10 U/L) to true (automated) results (U/L) is shown in Figure 2B. There were three devices for which visual-read results (from both nurses) fell in Bin 1, but true results were in Bin 3 (we refer to these as “major errors,” given that this would mean that clinically significant hepatotoxicity [>200 U/L] would have been missed) (Figure 2B). All three of these devices were from Lot 1. Other than this finding, there were no other notable differences in accuracy between the two device lots. Reanalysis of the bin placement results (Figure 2C) using a binary cutoff (<120 U/L = low, ≥120 U/L = high), as might be used in a “triage” or “screening” use scenario, generated bin placement accuracies of 88.0/86.3% for the “low” bin for Nurse 1 and Nurse 2, respectively, and 73.3/80.0% for the “high” bin.

#### Inter-reader agreement

**Figure 3:**
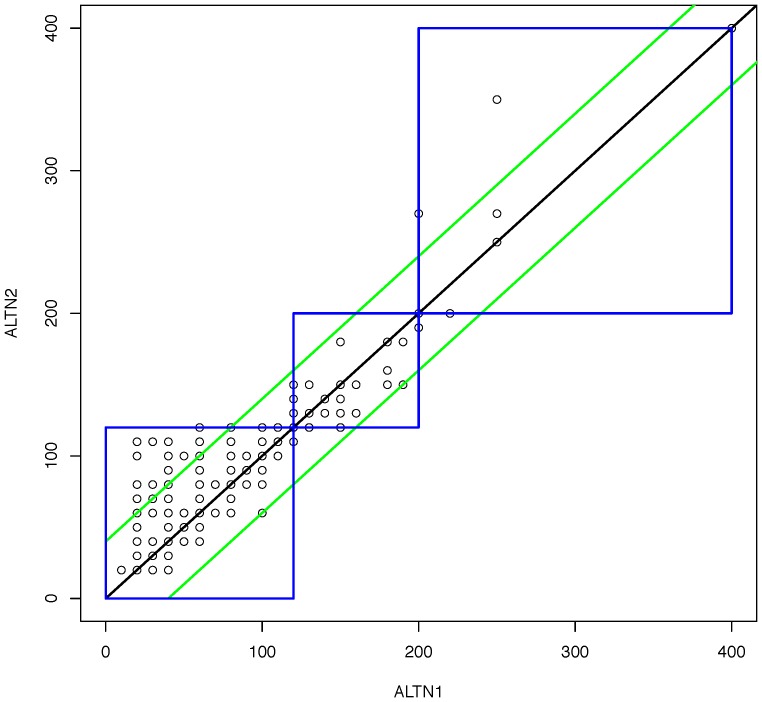
Analysis of inter-reader agreement. Visual ALT results recorded by Nurse 1 (ALTN1) were plotted against visual ALT results recorded by Nurse 2 (ALTN2) for each device to evaluate inter-reader agreement. The black diagonal line corresponds to the line of equality; the green diagonal lines correspond to +/−40 U/L from the line of equality; and the blue boxes represent ALT bins within which values for both readers are within the same range: <3x ULN (0–119 U/L), 3 to 5x ULN (120–200 U/L), or >5x ULN (>200 U/L). (ALT = alanine aminotransferase. ULN = upper limit of normal).

Nurse 1 and Nurse 2 agreed with each other regarding bin placement for 96.3% (95% C.I., 94.3–97.7) of device results. This high level of agreement between two readers evaluating the same device in real time is graphically represented in Figure 3, which plots the ALT result (to the nearest 10 U/L) read by Nurse 1 against the ALT result read by Nurse 2 for each device (of note, the high level of agreement between Nurse 1 and Nurse 2 visual ALT reads is also evident when comparing the two plots in Figure 2B). An intraclass correlation coefficient of 0.89 (95% CI: 0.87–0.91) also indicated high consistency for semi-continuous results (U/L). As noted above, Nurse 1 and Nurse 2 also agreed with each other on 95.6% of device validity classifications.

## Discussion

Paper-based microfluidic devices offer the potential to meet the need for low-cost, high-quality diagnostics that can improve patient care in resource-limited settings. This study is the first-ever field study performed with a patterned paper-based microfluidic device and, we believe, establishes this paper-based transaminase test as the most advanced representative of this emerging class of devices. Our findings of extremely high inter-operator agreement for visual reading–obtained in a target clinical population and environment, as performed by local health care workers–indicate that the device operation and reading process is both feasible and reproducible, thus answering a major question about the potential usability of this type of device. Bin placement accuracy data and lot-to-lot variability analysis have identified specific targets for device optimization and material quality control, and experiments to address both aspects are currently in progress in preparation for further field testing.

Successful development of this device for clinical use will require further iterative device optimization, thorough exploration of manufacturing conditions, device stability, and material quality control, and further field testing under varied environmental conditions. The intensive training allowed in the context of this first field study is unlikely to be feasible or reproducible outside of study contexts. A thorough understanding of the minimal training requirements for novice users will ultimately be key to understanding the range of clinical environments in which this test can be used–whether that be in centralized clinics as performed by trained staff, decentralized clinical settings as performed by minimally trained health-care workers, or even at home as performed by patients themselves.

Our study does have some limitations which should be noted here. First, while the clinic nurses were not told anything about prior ALT data for any subject, it is possible that they may have known some of the patients from prior clinic visits and may have had some recollection of their baseline ALT status. Second, because our protocol was designed to have no impact on routine clinical care, secondary laboratory data were only available when they were included as part of routine patient care. We were therefore unable to obtain comprehensive data for HCV or HBV status (as many patients had not had prior testing), or for other lab tests done concurrently with ALT, such as hematocrit, hemoglobin, platelet count, or creatinine (as many patients did not have these tests ordered for their routine clinical care), so were unable to assess impact of these variables on test results. In the subset of subjects whose providers ordered AST testing, elevated AST levels did not appear to impact the accuracy of the paper-based ALT test, supporting our prior findings that AST did not interfere with the ALT test [3]. Third, we note that the lower limit of detection (LLOD) of the paper-based test (calculated using scanned images of results obtained using spiked artificial plasma buffer, [3]) is 53 U/L, whereas the LLOD of the automated test is 1 U/L. We did allow the nurses to call visual results below this formally calculated LLOD, given that it may be possible to visually discriminate color changes in this lower range ([3], Fig. 1C). However, based on this formal LLOD, it would not reliably be possible to distinguish between completely normal (<40 U/L) and mildly elevated ALT (e.g. 53 U/L) with the paper-based test as currently designed. Finally, we note the issue of disagreement between readers (both those reading together in real time, and those reading scanned images) in visual interpretation of the presence or absence of hemolysis in the negative control zone, which in turn impacts interpretation of device validity. While the frequency of visibly detectable hemolysis will be addressed by material quality control, the questions regarding potential variability in the detection and interpretation of hemolysis remain and will require additional attention in future field studies.

The clinical diagnostic application chosen for initial demonstration of this paper-based microfluidic platform has global relevance. Hepatotoxicity is a major adverse event associated with both HIV and TB therapy, and monitoring for DILI is critically important in the care of these patients. Hepatotoxicity rates associated with nevirapine-based HIV therapy (widely used in the developing world, including our study site) can exceed 13%, depending on treatment course and underlying risk factors [2], [23], [24]. The overall incidence of clinically relevant hepatotoxicity on TB therapy (typically due to isoniazid, rifampin, and/or pyrazinamide) ranges from 2 to 33%, and risk may be increased by multiple factors, including hepatitis (B and/or C), alcohol use, and increasing age [1], [25]. Simultaneous treatment for both TB and HIV can generate additive risk of hepatotoxicity [26], [27]. Worldwide, hundreds of other commonly-used drugs have been associated with hepatotoxicity, and the scientific quest for greater understanding of DILI pathophysiology and susceptibility continues [28]–[30]. In practice, however, it remains difficult to predict which patients on treatment will actually develop hepatotoxicity [31], and thus it is essential to actively monitor at-risk patients to detect DILI.

There are two FDA-approved devices that could potentially be used for rapid POC testing, Roche Reflotron® Plus (Roche Diagnostics, Indianapolis, IN) and Cholestech LDX® (Alere, San Diego, CA), but both are currently off the market in the United States. Moreover, both are relatively expensive (US$3,000–6,000 for the reader and approximately US$4 per test) and rely on complex electronics and electricity/battery. While manufacturing costs for this device are difficult to calculate accurately *a priori*, given dependence on several key variables (including location of manufacturing), DFA anticipates that the device can ultimately be produced at a cost of less than US$0.10 per test.

An important question to consider as the device is further optimized for ultimate clinical use is which ALT bin cutoffs would have the highest utility worldwide given existing country- and disease-specific clinical management guidelines. Thus far, this test has been optimized for detection of ALT values >3x and >5x ULN, given that US TB treatment guidelines [1] emphasize these cutoffs (in concert with symptoms of hepatotoxicity) for making management decisions. HIV treatment guidelines in the United States [2] do not recommend strict ALT (or AST) cutoffs for clinical management of DILI, but do note that some experts recommend discontinuing drug treatment when the ALT level rises to more than 5–10x ULN. The World Health Organization (WHO) guidelines for HIV treatment [32] use a grading scale for ALT (and AST) that is identical to the AIDS Clinical Trial Group (ACTG) adverse event scale [33], with cutoffs at 2.5x, 5x, and 10x ULN. Minor ALT elevations (defined as less than 5x ULN) can be managed with observation, and treatment can be continued. Elevations above 5x ULN, however, prompt discontinuation of ART. Many developing countries, including Vietnam, South Africa, and India, have adopted the WHO grading scale for liver function monitoring in their national HIV treatment guidelines [22], [34], [35]. While we hope that optimized visual resolution in the 3–5x ULN range will allow device utility across these varied guidelines, we are also evaluating a possible “triage” use scenario, in which paper-based test values above a pre-specified threshold would prompt automated quantitative testing (by venipuncture). Performance of the device at various bin cutoffs will continue to be closely monitored in future field studies.

An additional question is whether the final version of the device should include only an ALT test, or both AST and ALT tests as in the prototype tested previously [3]. While most clinicians in the United States tend to order both ALT and AST measurements, US guidelines for monitoring for DILI during treatment of TB [1] focus recommendations on ALT levels, while also noting that AST can sometimes provide adjunctive information (e.g., alcohol-related transaminitis); WHO guidelines [36] mention only ALT. Many international HIV treatment guidelines recommend only ALT for routine monitoring for DILI in patients on ART. The Southern African HIV Clinicians Society explicitly cites cost considerations in their recommendation to use only ALT measurement for DILI monitoring [34]. The WHO and Vietnam national HIV treatment guidelines similarly recommend only ALT monitoring (but without giving any underlying rationale) [22], [32].

Because this paper-based microfluidic transaminase test is the first device of its class to come this far down the pathway towards clinical use, there are no clear precedents for performance standards–each aspect of its design and performance is effectively being evaluated for the first time. Even the question “how accurate must this device be in order to be clinically useful?” does not have a simple answer, and we anticipate that this discussion will play a large role in the regulatory approval process for this device and other similar devices that follow. Ideally, this device would simply be just as accurate as automated testing performed on blood obtained by venipuncture. However, it can be argued that it is unreasonable to expect the performance of a paper-based test to exactly match that of an automated test platform. The benefits of extremely low cost, simplicity, and POC use may ultimately allow a slightly less accurate paper-based test to have higher overall clinical impact than a more accurate but expensive and technically complicated test not available at the POC.

In conclusion, our study provides significant momentum to the rapidly expanding field of paper-based microfluidics and advances us towards the goal of providing universal access to POC screening for DILI. We anticipate that this device, once development is complete, will make extremely inexpensive and minimally invasive transaminase testing available at POC for all who need it, providing distinct advantages over current automated methods using venipuncture. Given that aversion to venipuncture can be a barrier to optimal care [37], this fingerstick test could conceivably also improve treatment adherence. Finally, this work opens the door to development of similar paper-based assays for other clinically important analytes.

## References

[1] Saukkonen JJ, Cohn DL, Jasmer RM, Schenker S, Jereb JA, et al (2006) An official ATS statement: hepatotoxicity of antituberculosis therapy. Am J Respir Crit Care Med 174: 935–952. 17021358 10.1164/rccm.200510-1666ST

[2] Panel on Antiretroviral Guidelines for Adults and Adolescents (2012) Guidelines for the use of antiretroviral agents in HIV-1-infected adults and adolescents. Department of Health and Human Services: 1–161. Available: http://www.aidsinfo.nih.gov/ContentFiles/AdultandAdolescentGL.pdf. Accessed 2012 Dec 28.

[3] Pollock NR, Rolland JP, Kumar S, Beattie PD, Jain S, et al (2012) A paper-based multiplexed transaminase test for low-cost, point-of-care liver function testing. Sci Transl Med 4: 152ra129. 10.1126/scitranslmed.3003981 PMC3624093 22993296

[4] Martinez AW, Phillips ST, Whitesides GM, Carrilho E (2010) Diagnostics for the developing world: microfluidic paper-based analytical devices. Anal Chem 82: 3–10. 20000334 10.1021/ac9013989

[5] Martinez AW, Phillips ST, Carrilho E, Thomas SW (2008) Simple telemedicine for developing regions: camera phones and paper-based microfluidic devices for real-time, off-site diagnosis. Anal Chem 80: 3699–3707. 18407617 10.1021/ac800112r PMC3761971

[6] Martinez AW, Phillips ST, Butte MJ, Whitesides GM (2007) Patterned paper as a platform for inexpensive, low-volume, portable bioassays. Angew Chem Int Ed Engl 46: 1318–1320. 17211899 10.1002/anie.200603817 PMC3804133

[7] Martinez AW, Phillips ST, Whitesides GM (2008) Three-dimensional microfluidic devices fabricated in layered paper and tape. Proc Natl Acad Sci U S A 105: 19606–19611. 19064929 10.1073/pnas.0810903105 PMC2604941

[8] Cheng CM, Martinez AW, Gong J, Mace CR, Phillips ST, et al (2010) Paper-based ELISA. Angew Chem Int Ed Engl 49: 4771–4774. 20512830 10.1002/anie.201001005

[9] Nie Z, Deiss F, Liu X, Akbulut O, Whitesides GM (2010) Integration of paper-based microfluidic devices with commercial electrochemical readers. Lab Chip 10: 3163–3169. 20927458 10.1039/c0lc00237b PMC3060706

[10] Osborn JL, Lutz B, Fu E, Kauffman P, Stevens DY, et al (2010) Microfluidics without pumps: reinventing the T-sensor and H-filter in paper networks. Lab Chip 10: 2659–2665. 20680208 10.1039/c004821f PMC4892122

[11] Fu E, Lutz B, Kauffman P, Yager P (2010) Controlled reagent transport in disposable 2D paper networks. Lab Chip 10: 918–920. 20300678 10.1039/b919614e PMC3228840

[12] Lutz BR, Trinh P, Ball C, Fu E, Yager P (2011) Two-dimensional paper networks: programmable fluidic disconnects for multi-step processes in shaped paper. Lab Chip 11: 4274–4278. 22037591 10.1039/c1lc20758j PMC4892121

[13] Fu E, Kauffman P, Lutz B, Yager P (2010) Chemical signal amplification in two-dimensional paper networks. Sens Actuators B Chem 149: 325–328. 20706615 10.1016/j.snb.2010.06.024 PMC2917776

[14] Khan MS, Thouas G, Shen W, Whyte G, Garnier G (2010) Paper diagnostic for instantaneous blood typing. Anal Chem 82: 4158–4164. 20415489 10.1021/ac100341n

[15] Li X, Tian J, Garnier G, Shen W (2010) Fabrication of paper-based microfluidic sensors by printing. Colloids Surf B Biointerfaces 76: 564–570. 20097546 10.1016/j.colsurfb.2009.12.023

[16] Dungchai W, Chailapakul O, Henry CS (2010) Use of multiple colorimetric indicators for paper-based microfluidic devices. Anal Chim Acta 674: 227–233. 20678634 10.1016/j.aca.2010.06.019

[17] Fenton EM, Mascarenas MR, Lopez GP, Sibbett SS (2009) Multiplex lateral-flow test strips fabricated by two-dimensional shaping. ACS Appl Mater Interfaces 1: 124–129. 20355763 10.1021/am800043z

[18] Vella SJ, Beattie P, Cademartiri R, Laromaine A, Martinez AW, et al (2012) Measuring markers of liver function using a micropatterned paper device designed for blood from a fingerstick. Anal Chem 84: 2883–2891. 22390675 10.1021/ac203434x PMC3320108

[19] Li X, Ballerini DR, Shen W (2012) A perspective on paper-based microfluidics: Current status and future trends. Biomicrofluidics 6: 11301–1130113. 22662067 10.1063/1.3687398 PMC3365319

[20] Delaney JL, Hogan CF, Tian J, Shen W (2011) Electrogenerated chemiluminescence detection in paper-based microfluidic sensors. Anal Chem 83: 1300–1306. 21247195 10.1021/ac102392t

[21] Chin CD, Linder V, Sia SK (2012) Commercialization of microfluidic point-of-care diagnostic devices. Lab Chip 12: 2118–2134. 22344520 10.1039/c2lc21204h

[22] Vietnam Ministry of Health (2009) Guidelines on the Diagnosis and Treatment of HIV/AIDS. Hanoi.

[23] McKoy JM, Bennett CL, Scheetz MH, Differding V, Chandler KL, et al (2009) Hepatotoxicity associated with long- versus short-course HIV-prophylactic nevirapine use: a systematic review and meta-analysis from the Research on Adverse Drug events And Reports (RADAR) project. Drug Saf 32: 147–158. 19236121 10.2165/00002018-200932020-00007 PMC2768573

[24] Martinez E, Blanco JL, Arnaiz JA, Perez-Cuevas JB, Mocroft A, et al (2001) Hepatotoxicity in HIV-1-infected patients receiving nevirapine-containing antiretroviral therapy. AIDS 15: 1261–1268. 11426070 10.1097/00002030-200107060-00007

[25] Tostmann A, Boeree MJ, Aarnoutse RE, de Lange WC, van der Ven AJ, et al (2008) Antituberculosis drug-induced hepatotoxicity: concise up-to-date review. J Gastroenterol Hepatol 23: 192–202. 17995946 10.1111/j.1440-1746.2007.05207.x

[26] Shipton LK, Wester CW, Stock S, Ndwapi N, Gaolathe T, et al (2009) Safety and efficacy of nevirapine- and efavirenz-based antiretroviral treatment in adults treated for TB-HIV co-infection in Botswana. Int J Tuberc Lung Dis 13: 360–366. 19275797 PMC2696339

[27] Hoffmann CJ, Charalambous S, Thio CL, Martin DJ, Pemba L, et al (2007) Hepatotoxicity in an African antiretroviral therapy cohort: the effect of tuberculosis and hepatitis B. AIDS. 21: 1301–1308. 10.1097/QAD.0b013e32814e6b08 17545706

[28] Lee WM (1995) Drug-induced hepatotoxicity. N Engl J Med 333: 1118–1127. 7565951 10.1056/NEJM199510263331706

[29] Grant LM, Rockey DC (2012) Drug-induced liver injury. Curr Opin Gastroenterol 28: 198–202. 22450893 10.1097/MOG.0b013e3283528b5d

[30] Davern TJ (2012) Drug-induced liver disease. Clin Liver Dis 16: 231–245. 22541696 10.1016/j.cld.2012.03.002

[31] Centers for Disease Control and Prevention (2010) Severe isoniazid-associated liver injuries among persons being treated for latent tuberculosis infection–United States, 2004–2008. MMWR Morb Mortal Wkly Rep 59: 224–229. 20203555

[32] World Health Organization (2010) Antiretroviral therapy for HIV infection in adults and adolescents in resource-limited settings: recommendations for a public health approach, 2010 revision. Geneva, Switzerland. 23741771

[33] AIDS Clinical Trials Group (1996)Table of grading severity of adult adverse experiences. Rockville, MD: Division of AIDS, National Institute of Allergy and Infectious Diseases.

[34] Meintjes G, Maartens G, Boulle A, Conradie F, Goemaere E, et al (2012) Guidelines for antiretroviral therapy in adults. S Afr J HIV Med 13: 114–133.

[35] National AIDS Control Organisation (2007) Antiretroviral therapy guidelines for HIV-infected adults and adolescents including post-exposure prophylaxis. India Ministry of Health and Family Welfare.

[36] World Health Organization (2009) Treatment of tuberculosis guidelines 4th edition. Geneva, Switzerland: World Health Organization; 2009.

[37] Shieh FK, Snyder G, Horsburgh CR, Bernardo J, Murphy C, et al (2006) Predicting non-completion of treatment for latent tuberculous infection: a prospective survey. Am J Respir Crit Care Med 174: 717–721. 16809632 10.1164/rccm.200510-1667OC

